# Genome-Wide Association Studies of Live Weight at First Breeding at Eight Months of Age and Pregnancy Status of Ewe Lambs

**DOI:** 10.3390/genes14040805

**Published:** 2023-03-27

**Authors:** Emmanuelle Haslin, Emma J. Pettigrew, Rebecca E. Hickson, Paul R. Kenyon, Kristene R. Gedye, Nicolas Lopez-Villalobos, J. M. D. R. Jayawardana, Stephen T. Morris, Hugh T. Blair

**Affiliations:** 1 School of Agriculture and Environment, Massey University, Palmerston North 4442, New Zealand; p.r.kenyon@massey.ac.nz (P.R.K.); n.lopez-villalobos@massey.ac.nz (N.L.-V.); d.jayawardana@massey.ac.nz (J.M.D.R.J.); s.t.morris@massey.ac.nz (S.T.M.); h.blair@massey.ac.nz (H.T.B.); 2 Wairere Ltd., Masterton 5882, New Zealand; epettigrew1@gmail.com; 3 Focus Genetics, Napier 4144, New Zealand; rebecca.hickson@focusgenetics.com; 4 School of Veterinary Science, Massey University, Palmerston North 4442, New Zealand; k.gedye@massey.ac.nz; 5 Department of Animal Science, Faculty of Animal Science and Export Agriculture, Uva Wellassa University, Badulla 90000, Sri Lanka

**Keywords:** genomic heritability, growth, candidate genes, fertility

## Abstract

This study estimated genetic parameters and identified candidate genes associated with live weight, and the occurrence of pregnancy in 1327 Romney ewe lambs using genome-wide association studies. Phenotypic traits considered were the occurrence of pregnancy in ewe lambs and live weight at eight months of age. Genetic parameters were estimated, and genomic variation was assessed using 13,500 single-nucleotide polymorphic markers (SNPs). Ewe lamb live weight had medium genomic heritability and was positively genetically correlated with occurrence of pregnancy. This suggests that selection for heavier ewe lambs is possible and would likely improve the occurrence of pregnancy in ewe lambs. No SNPs were associated with the occurrence of pregnancy; however, three candidate genes were associated with ewe lamb live weight. Tenascin C (*TNC*), TNF superfamily member 8 (*TNFSF8*) and Collagen type XXVIII alpha 1 chain (*COL28A1*) are involved in extracellular matrix organization and regulation of cell fate in the immune system. *TNC* may be involved in ewe lamb growth, and therefore, could be of interest for selection of ewe lamb replacements. The association between ewe lamb live weight and *TNFSF8* and *COL28A1* is unclear. Further research is needed using a larger population to determine whether the genes identified can be used for genomic selection of replacement ewe lambs.

## 1. Introduction

Farmers can increase farm profitability by breeding their ewe lambs at approximately eight months of age as it can increase the number of lambs born per ewe in her lifetime and per farm per year, thus increasing income [1,2]. There is a positive phenotypic relationship between the likelihood of pregnancy occurring at this age (fertility) and live weight of ewe lambs at breeding at eight months of age [1,3]. The potential genetic improvement of these two traits is dependent on their heritability and genetic correlation. Previous studies reported that the heritability of live weight of lambs at nine months of age ranged from 0.17–0.41 [4,5,6,7,8], and the heritability of ewe lamb fertility (defined as pregnant or not) was 0.09 [4]. In the same study, a negative genetic correlation (−0.25) was reported between ewe lamb fertility and ewe lamb live weight at three months of age [4], indicating that a genetically heavier ewe at three months of age would be less likely to get pregnant as an ewe lamb. Based on this, selecting for just one trait will likely have a negative influence on the other. However, previous studies have also reported that heritability of live weight increases with age, likely due to the reduction of the maternal effect [6,8,9]. The genomic relationship between live weight at breeding (approximately eight months of age) and fertility in ewe lambs is currently unknown.

A potential selection tool is the use of single-nucleotide polymorphisms (SNPs). With genomic technologies becoming cost-effective, selection of replacements via genomic information is a realistic tool for farmers to use and can be used to select replacement animals at an earlier age [10,11]. The use of genomic breeding values in replacement selection has been projected to increase the rate of genetic gain in the sheep industry by 22–54% [12]. Single nucleotide polymorphisms (SNPs) occur across entire genomes and are locations where a single nucleotide or base changes [13,14,15]. Some SNPs have significant associations with phenotypic traits, indicating that the genome region they are part of, or are near to, has an impact on the expression of the trait [13,14]. Both pregnancy occurrence and live weight at the start of breeding are complex traits likely involving many genes directly and indirectly influencing their phenotypic expression [15]. An ewe lamb needs to attain puberty prior to the breeding period. The attainment of puberty, and the regulation of the estrous cycle have similar genetic and hormonal control [16], and, therefore, genes associated with the attainment of puberty can potentially be used as a proxy for pregnancy occurrence. However, the attainment of puberty is also influenced by live weight of ewe lambs, which has medium heritability [5,6,8], indicating that at least a fraction of this trait is under genetic control. Therefore, identifying genes associated with live weight of ewe lambs at eight months of age could be important when selecting ewe lamb replacements.

The aim of this study was to perform genome-wide association analyses to estimate the genetic parameters and identify candidate genes associated with live weight at eight months of age, and the occurrence of pregnancy in Romney ewe lambs, when bred for the first time, at eight months of age. It was hypothesized that the genetic correlation between live weight at eight months and fertility in ewe lambs would be positive.

## 2. Materials and Methods

The experiment was conducted at Massey University’s Riverside farm (latitude: 40°50′35″ S, longitude: 175°37′55″ E), 11 km north of Masterton, New Zealand, from January 2017 to July 2018, with the approval of the Massey University Animal Ethics Committee (MUAEC 17/16 and MUAEC 17/12).

### 2.1. Animals and Phenotypes

Eight groups of Romney ewe lambs born in either 2016 or 2017 and introduced to rams for the first time at eight months of age were included in this study. Ewe lambs born in 2016 and 2017 were also included in separate studies from which phenotypic information were collected; [17,18] for 2016-born, and [3,19,20] for 2017-born ewe lambs. The 2016-born ewe lambs were born to either recorded (Gen) or unrecorded (Com) mature dams and grazed on pasture in either group A (*n* = 513) or B (*n* = 513), making up four contemporary groups: 16GenA, 16ComA, 16GenB and 16ComB. The 2017-born ewe lambs (*n* = 493) were born to either mature ewes (Mat) or ewe lambs (Lamb). Those born to mature ewes were either fed concentrates and pasture between weaning and their first breeding (Pref), or fed only pasture (Norm), whilst those born to ewe lambs were all fed concentrates and pasture and were either single- (Sing) or twin-born (Twin). This resulted in four additional contemporary groups: 17MatNorm, 17MatPref, 17LambSing and 17LambTwin.

Ewe lambs were bred with mature rams for 34 days. The 2016-born ewe lambs were joined with 18 rams in each group A and B. Group A and B weighed on average 46.2 kg ± 0.18 and 48.3 kg ± 0.19 at the start of breeding, respectively. The 2017-born lambs were bred with 12 rams with all ewe lambs in a single cohort and weighed on average 43.7 kg ± 0.25 at the start of breeding. The minimum live weight to be presented for breeding was 38 kg for the 2016-born ewe lambs [17] and 39 kg for the 2017-born ewe lambs [3,20]. Ewe lambs that did not reach the minimum live weight threshold were removed from their respective groups during the breeding period.

The first phenotypic trait considered was live weight at the start of the breeding period at eight months of age. All ewe lambs with a live weight recorded at the start of breeding were included in the analysis. The second phenotypic trait considered in the analysis was occurrence of pregnancy during the 34-day breeding period. Occurrence of pregnancy (Pregnant or Not pregnant) was determined and collected at 93 and 84 days after the introduction to the rams via transabdominal ultrasound for the 2016- and 2017-born ewe lambs, respectively. To enable consistent analysis, all ewe lambs that were lighter than 39 kg at the start of breeding were considered as not exposed to a ram (*n* = 9 in 2016-born and *n* = 98 in 2017-born). Therefore, there were three possible pregnancy outcomes: pregnant, non-pregnant after exposure to the ram, and not exposed to the ram. Ewes not exposed to the ram were not included in the analysis of occurrence of pregnancy.

Out of the final population of 1327 ewe lambs used in the analysis, 712 had both parents known, 494 had one parent unknown (484 unknown sire and 10 unknown dam) and 121 had both parents unknown based on DNA parentage and data collected during the lambing periods.

### 2.2. Single-Nucleotide Polymorphism Analysis

Ear tissue samples were collected from each of the 1519 ewe lambs for DNA isolation. Ewe lambs were genotyped using a 15,000 SNP chip (Infinium HTS iSelect customized at 15,000 SNP) and using a custom Infinium Array (Illumina, San Diego, CA, USA) designed by Equine Parentage and Animal Genetics Service Centre (Massey University, Palmerston North, New Zealand; [21]). Ewe lambs were removed for call rates lower than 95%, or for not having both phenotypic and genotypic records, resulting in a total population of 1327 ewe lambs with a recorded phenotype and a viable SNP profile (Table 1). Loci with a call rate ≤80% or minor allele frequency ≤0.05 were excluded from the dataset. A total of 13,500 SNPs were available for association analysis. The contemporary groups for all ewe lambs are presented in Table 1.

The map positions of the SNPs were based on ovine genome assembly (Rambouillet_v1.0) produced by the International Sheep Genome Consortium (ISGC). The average distance between SNPs was 304,834 bp.

### 2.3. Statistical Analysis

Variance components, heritability and correlations were estimated using ASReml (v 3.0) [22]. Variance components were estimated using a single-trait animal model represented as follows:

y=Xb+Za+e

where *y* is the vector of observations for phenotypic traits; *b* is the vector of fixed effect of contemporary group, *a* is the vector of additive genetic effects, *e* is the vector of random residual effects. *X* and *Z* are incidence matrices relating to the records of fixed and animal additive genetic effects, respectively. The expected values (*E*) of the variables were assumed as *E*(*y*) = *Xb*, *E*(*a*) = 0 and *E*(*e*) = 0. The residuals were assumed to be independently distributed and var(*y*) =${\mathrm{ZAZ}}^{\prime }A\sigma_{g}^{2}+R$, var(*a*) = A$\sigma_{g}^{2}$ and var(*e*) = *I*$\sigma_{e}^{2}$= R; where $\sigma_{g}^{2}$ is the animal additive genetic variance, $\sigma_{e}^{2}$ is the random residual variance, A is the numerator relationship matrix between all the ewes and I is the identity matrix which corresponds to the number of ewes with records.

The heritability $\left(h^{2}\right)$ estimate was calculated as [23],

h2=σg2σg2+σe2

(Co)variance components and genetic and phenotypic correlations were also estimated using a bivariate animal model. In matrix notation, the bivariate model was represented as follows:

$$\left[\begin{array}{c} y_{1}\\ y_{2} \end{array}\right]=\left[\begin{array}{cc} X_{1} & 0\\ 0 & X_{2} \end{array}\right]\left[\begin{array}{c} b_{1}\\ b_{2} \end{array}\right]+\left[\begin{array}{cc} Z_{1} & 0\\ 0 & Z_{2} \end{array}\right]\left[\begin{array}{c} a_{1}\\ a_{2} \end{array}\right]+\left[\begin{array}{c} e_{1}\\ e_{2} \end{array}\right]$$

where $y_{1}$ and $y_{2}$ are the vectors of phenotypic measures for two traits; $b_{1}$ and $b_{2}$ are the vectors of fixed effect for contemporary group; $X_{1}$, $X_{2}$, $Z_{1}$ and $Z_{2}$ are design matrices relating the fixed and animal additive genetic effects related to the $y_{1}$ and $y_{2}$ phenotypes, respectively; $a_{1}$ and $a_{2}$ are the vectors of random effects of animal for each trait; and $e_{1}$ and $e_{2}$ are vectors of residual errors. The expected values of the variables were assumed to be *E*($y_{1}$) = $X_{1}b_{1}$; *E*($y_{2}$) = $X_{2}b_{2}$; *E*(a) = 0 and *E*(*e*) = 0. The residuals were assumed to be normally distributed with zero mean and the following co(variance) structure:

$$var\left[\begin{array}{c} \begin{array}{c} a_{1}\\ a_{2} \end{array}\\ e_{1}\\ e_{2} \end{array}\right]=\left[\begin{array}{ccc} A\sigma_{a1}^{2} & A\sigma_{a12} & \begin{array}{cc} 0 & 0 \end{array}\\ A\sigma_{a12} & A\sigma_{a2}^{2} & \begin{array}{cc} 0 & 0 \end{array}\\ \begin{array}{c} 0\\ 0 \end{array} & \begin{array}{c} 0\\ 0 \end{array} & \begin{array}{cc} \begin{array}{c} {I\sigma }_{e1}^{2}\\ I\sigma_{e12} \end{array} & \begin{array}{c} I\sigma_{e12}\\ I\sigma_{e2}^{2} \end{array} \end{array} \end{array}\right]$$

where $\sigma_{a1}^{2}$ is the additive genetic variance of the trait 1; $\sigma_{a2}^{2}$ is the additive genetic variance of trait 2 and $\sigma_{a12}$ is the additive genetic covariance between both traits. I is an identity matrix with number of ewes with records; $\sigma_{e1}^{2}$ is the residual variance for trait 1; $\sigma_{e2}^{2}$ is the residual variance of trait 2 and $\sigma_{e12}$ is the residual covariance between both traits.

Genetic correlations ($r_{g}$) were estimated as [23]:

$$r_{g}=\frac{\sigma_{a12}}{\sigma_{a1}\times \sigma_{a2}}$$

where $\sigma_{a1}$ and $\sigma_{a2}$ were genetic additive standard deviation for trait 1 and trait 2, respectively.

Phenotypic correlations ($r_{P}$) were estimated as [23]:

$$r_{P}=\frac{\sigma_{P12}}{\sigma_{P1}\times \sigma_{P2}}$$

where $\sigma_{P12}$ is phenotypic covariance between phenotypic trait 1 and trait 2; $\sigma_{P1}$ and $\sigma_{P2}$ are phenotypic standard deviation for trait 1 and trait 2, respectively.

A single SNP genome-wide association study (GWAS) was performed using a mixed linear model in the software package GCTA [24]. The statistical model was defined as follows:

(1) y=µ+Xβ+Zu+g+e

where *y* is the vector of the phenotypes; *µ* is the vector of the overall mean; *β* is the vector of fixed effect of contemporary group as described in Table 1; X is the incidence matrix of *β* to *y*; *u* is the vector of the additive effect of the candidate SNP to be tested for association; *Z* is the vector of the genotypes for the SNP, coded as 0, 1 or 2; g is the vector of polygenic effects with $g\sim N(0,GRM\sigma_{g}^{2}),$ where *GRM* denotes the genomic relationship matrix among animals and $\sigma_{g}^{2}$ is the additive genetic variance; and e is the vector of random residual errors with $e\sim N(0,I\sigma_{e}^{2})$, where *I* is the identity matrix of size of 1327 and $\sigma_{e}^{2}$ is residual variance. Genomic heritability estimate was calculated for each phenotypic trait using variance components from REML option in GCTA for 13,500 SNPs data.

The level of a significant association between a marker and the phenotype was adjusted using a Bonferroni correction to account for multiple SNP comparisons. The Bonferroni corrected *p*-value threshold for genome-wide significance level was 0.05/number of SNPs = 3.70 × $10^{-6}$, which corresponded to 5.43 in −log_10_ (*p*-value) scale. However, this correction has been considered too conservative, therefore, a *p*-value threshold for suggestive linkage was also computed. This suggestive linkage procedure corresponded one false positive result across the genome and less conservatively, accordingly, Lander and Kruglyak [25] proposed the following adjustment of nominal *p*-value one, which was (1/number of SNPs = 7.54 × $10^{-5}$) that correspond to 4.12 in −log_10_ (*p*-value) scale.

The estimated associations were represented in Manhattan plots in which −log_10_ (*p*-value) were plotted against the genomic locations of the markers using the qqman package in R software 4.2.1 [26].

### 2.4. Candidate Genes and Functional Analysis

Each SNP that was significant or nearly significant was examined to locate the genes within 150 kb upstream and downstream from the SNP. Candidate genes were identified for significant SNPs using the *Ovis Aries* reference genome in the Ensembl database (Rambouillet_v1.0; http://asia.ensembl.org/Ovis_aries_rambouillet/Info/Index, accessed on 3 November 2022). If no annotated genes were present in the 300 kb examined area, a blastn was performed in NCBI (https://blast.ncbi.nlm.nih.gov/Blast.cgi, accessed on 14 November 2022) on a nucleotide sequence including 500 bp around the significant SNP to identify a similar sequence in other species which has been annotated. The biological function of the genes was identified using PANTHER [27,28], and Reactome [29] was used to identify biological pathways.

## 3. Results

Ewe lambs that were pregnant after a 34-day breeding period represented 48.8% of the population and weighed 47.4 ± 0.15 kg at breeding, ewe lambs that were non-pregnant after exposure to the rams (43.2%) weighed 46.3 ± 0.17 kg at breeding, and ewe lambs that were not exposed to the rams (8.0%) weighed 35.5 ± 0.26 kg at breeding of their counterparts.

Heritability estimates for ewe lamb live weight and the occurrence of pregnancy were 0.57 and 0.31, respectively (Table 2). The pedigree-based heritability estimates were greater than the estimates obtained from the genomic relationship matrix (0.38 and 0.14, respectively; Table 2). The genetic and phenotypic correlations between the ewe lamb live weight and the occurrence of pregnancy were 0.19 and 0.69, respectively (Table 2).

**Figure 1:**
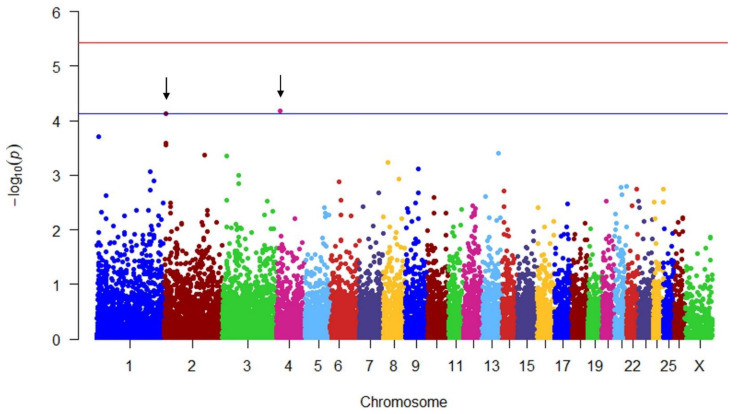
Manhattan plot showing the genome-wide association for live weight at breeding at eight months of age in ewe lambs. The red line includes the Bonferroni-adjusted genome-wide significant level at −log_10_ (*p* value) = 5.43, and the blue line includes the suggestive association significant level at −log_10_ (*p* value) = 4.12. Black arrows indicate significant single-nucleotide polymorphisms.

There were no significant (*p* > 0.05) regions associated with ewe lamb live weight at eight months of age, but two SNPs on chromosomes 2 and 4 were significant at a suggestive significance level (*p* < 7.54 × 10^−5^; Figure 1). Three candidate genes were identified to be associated with live weight of ewe lambs at eight months of age (Table 3 and Table 4), tenascin C (*TNC*), TNF superfamily member 8 (*TNFSF8*) and collagen type XXVIII α 1 chain (*COL28A1*).

**Figure 2:**
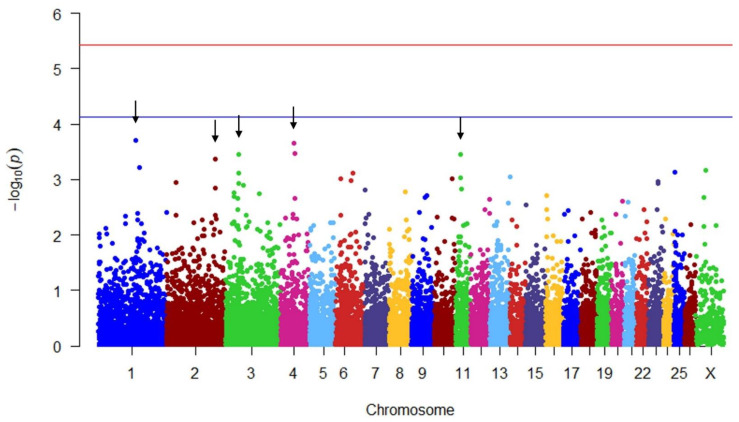
Manhattan plot showing the genome-wide association for the occurrence of pregnancy of ewe lambs when bred first at eight months of age The red line includes the Bonferroni-adjusted genome-wide significant level at −log_10_ (*p*-value) = 5.43, and the blue line includes the suggestive association significant level at −log_10_ (*p*-value) = 4.12. The black arrows indicate nearly significant peaks at suggestive significance level.

There were no significant (*p* > 0.05) regions associated with the occurrence of pregnancy in ewe lambs. Four peaks, indicating chromosomes 1, 2, 3, 4 and 11, were nearly significant at a suggestive significance level (*p* < 7.54 × 10^−5^; Figure 2), indicating trends of potential associations with the occurrence of pregnancy. No orthologs were identified for the SNP associated with occurrence of pregnancy on chromosome 3. The nearly significant SNPs associated with the occurrence of pregnancy are summarized in Table 3.

## 4. Discussion

### 4.1. Genetic Parameter Estimates

It was hypothesized that the genetic correlation between ewe lamb live weight at breeding and occurrence of pregnancy would be positive. In support of this, the phenotypic and genetic correlations between occurrence of pregnancy and ewe lamb live weight were indeed both positive. This indicated, in this study, that genetically heavier ewe lambs at eight months of age were more likely to get pregnant, and a selection for heavier ewe lambs at that age would increase the proportion of ewe lambs pregnant over time. This result contrasted with Fossceco and Notter [4], who reported a medium negative genetic correlation (−0.25) between live weight at three months of age and fertility in ewe lambs. Ewe lambs were bred at six months of age, at a minimum live weight of 27 kg, and were bred with ram lambs [4], both of which were reported to lower the reproductive performance of ewe lambs [1,37]. In addition, the live weight at three months of age used to estimate the genetic correlation with ewe lamb fertility included the live weight of both ewe and ram lambs [4], increasing the overall live weight in the estimation of the genetic correlation between these traits. These differences between studies likely impacted the genetic correlation between live weight at three months of age and ewe lamb fertility. Finally, the breed of ewe lambs could explain differences as Fossceco and Notter [4] used composite breeds including Dorset, Rambouillet and Finnish Landrace, whereas New Zealand Romney ewe lambs were used in this study.

The genomic heritability of both traits considered in this study was lower than pedigree-based heritability. A similar observation was reported by Khanzadeh, et al. [38] in dairy cows, whereas Almasi, et al. [5] reported consistent values between pedigree-based and genomic heritability of live weight in lambs at nine months of age (0.34 vs. 0.35, respectively). The genomic heritability reported in the same study [5] was similar to the genomic heritability of live weight of ewe lambs at eight months of age in the present study. In addition, the pedigree-based heritability of live weight at eight months of age (0.57) and occurrence of pregnancy in ewe lambs (0.31) were greater than what was reported in the literature (0.17–0.41 for live weight at nine months of age and 0.09 for fertility in ewe lambs [4,5,6,8]). The different breeds and environments of the sheep and the limited size of the ewe lamb population in the present study could explain these differences, in particular for binomial traits such as occurrence of pregnancy [6,9]. Pedigree-based heritabilities are likely to be overestimated compared to marker-based (genomic) heritability [38,39,40].

Combined, the results showed that selection of heavier ewe lambs at their first breeding at eight months of age is possible with medium genomic heritability. This selection on ewe lamb live weight should improve the occurrence of pregnancy in ewe lambs, leading to more pregnant ewe lambs in their first year of life and over time, and therefore, potentially greater ewe productivity. More research is needed to consolidate these results using a larger population of ewe lambs and higher density arrays to increase the accuracy of the genomic parameter estimations.

### 4.2. Candidate Genes

Knowledge of specific gene regions can be used to improve genomic approaches for selective breeding [15]. This can either be through marker-assisted selection to create genomically enhanced estimated breeding values (gEBVs) or by selecting for causal mutations where the function of a candidate gene and mutations are known [21,41]. Genomic predictors of genetic merit are especially beneficial in increasing the accuracy of prediction of those traits that are hard to measure at the time of replacement selection, such as meat quality traits and future reproductive traits [41].

Three candidate genes were associated with live weight of ewe lambs at the start of breeding at eight months of age: *TNC*, *TNFSF8* and *COL28A1*. Tenascin C (*TNC*) is expressed in neural, vascular and skeletal tissues during embryonic development [42], whereas in post-natal maturation, *TNC* is detected in the extracellular matrix in musculoskeletal tissues [32]. Gruber, et al. [32] showed that increased expression of *TNC* is involved in the adaptative response of musculoskeletal tissues to mechanical stress. At eight months of age, ewe lambs are still physically growing, which can be linked to mechanical stress for musculoskeletal cells and tissues [43,44,45]. There are complex reciprocal interactions between tissue mechanics and growth, involving feedback mechanisms [45]. It was reported that mechanical stress (e.g., compression or stretch) increase with development, making effective tissue growth slow down [45,46,47]. At this age, some ewe lambs would have attained puberty, indicating that they reached approximately 50 to 60% of their mature weight [48]. It could, therefore, be possible that the expression of *TNC* would be greater in ewe lambs that attained puberty at eight months of age, as they were more advanced in their growth and development.

The gene TNF superfamily member 8 (*TNFSF8*) is expressed in T cells and has a role in the regulation of cell survival and apoptosis [33,34]. In sheep, *TNFSF8* was mentioned by Lin, et al. [49] as potentially being associated with a parasite-infection response. *TNFSF8* was also upregulated by *IGF1* in cow embryos and was reported to improve the ability of cow embryos to develop pre-implantation [34]. A response in sheep similar to that of *TNFSF8* to *IGF1* in cows could be of great interest for ewe lamb replacement selection. *IGF1* is a well-accepted candidate gene impacting growth and development in sheep [50,51], in particular at nine months of age in Hulun Buir sheep [52]. The association between *TNFSF8* function and live weight of ewe lambs at eight months of age is not clear and requires further research.

Collagen type XXVIII α 1 chain (*COL28A1*) was reported to be expressed in the peripheral nervous system and myelin-free areas in mammals [35] and in muscles and skin in zebrafish, where it consists in four paralog genes and seems to be implicated in development [53]. This gene is detected and expressed in horse conceptuses 10 days post-ovulation and is associated with serine-type endopeptidase inhibitor activity [54], indicating a role in embryonal development. In sheep, *COL28A1* was found in sheep fetal heart exposed to hypoxia [55] and in multiparous non-pregnant and non-lactating ewes where it was associated with heat tolerance [56]. It was also suggested that collagen XXVIII could be involved in tissue repair processes [35]. Information about *COL28A1* is sparse across species, and its relationship with live weight or growth of ewe lambs is unknown. More research is required to determine its function in mammals and its relationship with ewe lamb live weight.

Combined, these results suggest that *TNC* and *TNFSF8* could be genes of importance in the growth and development of ewe lambs at eight months of age. Further investigations are warranted to determine their roles in ewe lamb development and whether they could be used for genetic selection to improve live weight of ewes at their first breeding at eight months of age.

No SNPs met the statistical threshold for association with occurrence of pregnancy in ewe lambs, but trends were observed on four chromosomes, and 15 potential genes were identified at these locations. Among these 15 potential genes, two were reported to have a link with fertility: solute carrier family 43 member 2 (*SLC43A2*) and tyrosine 3-monooxygenase/tryptophan-5-monooxygenase activation protein epsilon (*YWHAE*). *SLC43A2* is expressed in the endometrium, uterine epithelia and conceptus tissues in ewes [57], beef heifers [58] and mares [59]. The expression of *SLC43A2* increases during conceptus development in beef heifers [58] and, therefore, may be essential for conception and embryo development in ewe lambs. *YWHAE* is expressed in the ovaries, immature oocytes and mature eggs of mice [60]. Although oocyte-specific inactivation of this gene does not impact fertility or litter size, global inactivation (i.e., whole body) impairs in vivo fertility in mice [61]. Low expression of this gene could potentially reduce fertility in females. Although these genes were not statistically associated with occurrence of pregnancy in ewe lambs, they can be linked to fertility in females and, therefore, would likely benefit from further investigation.

### 4.3. Limitations

The threshold in live weight below which an ewe lamb was considered too light to be bred differed between years with 38 kg for the 2016-born ewe lambs and 39 kg for those born in 2017. For consistency in the analysis, ewe lambs weighing less than 39 kg were considered “not exposed to the rams” and therefore were not included in the estimation of the genetic parameters for the occurrence of pregnancy. Whilst ethically appropriate, not exposing lighter ewe lambs to the ram truncated the data and reduced the opportunity to identify highly fertile ewe lambs that became pregnant at a light weight. This limited the size of the ewe lamb population for the estimation of genetic parameters for the occurrence of pregnancy, and the identification of SNPs and candidate genes associated with the occurrence of pregnancy in ewe lambs.

None of the SNPs reached the genome-wide significant threshold for both ewe lamb live weight at eight months of age and occurrence of pregnancy. This could suggest that both traits are controlled by many genes, each having small effects. The limited size of the ewe lamb population and the low-density array used in this study challenged the detection of these potential small effects.

## 5. Conclusions

Ewe lamb live weight at eight months of age had medium genomic heritability, showing that selection for heavier ewe lambs could be integrated into the selection criteria for replacement animals. This selection on live weight would likely improve the occurrence of pregnancy in ewe lambs, resulting in an increase in ewe productivity. No genes were associated with the occurrence of pregnancy; however, *TNC*, *TNFSF8* and *COL28A1* were associated with ewe lamb live weight at eight months of age. Their functions are known to include extracellular matrix organization and regulation of cell survival and apoptosis in the immune system. *TNC*’s role suggests they may also be involved in the growth of ewe lambs and therefore could be of interest for replacement selection. The association between *TNFSF8* and *COL28A1* and ewe lamb live weight remains unclear. Further research is needed to consolidate these results using a larger population of ewe lambs. Investigations are also warranted to determine the specific roles of the genes identified in this study in ewe lamb development and whether they could be used for genomic selection to improve live weight of replacement ewe lambs, resulting in a corresponding increase in ewe lamb fertility through the positive genetic correlation.

## Figures and Tables

**Table 1:** Summary of the contemporary group effects used in the analysis of SNPs based on the year of birth, dam age group, birth rank, feed treatment and group. The *n* values presented were total numbers used in the analysis, with both phenotype and genotype recorded.

Contemporary Group	*n*	Year Born	Dam Age ^2^	Birth Rank	Feed Treatment
16GenA ^1^	371	2016	Mature ewes	Singletons and twins	Normal, Group A
16GenB ^1^	355	2016	Mature ewes	Singletons and twins	Normal, Group B
16ComA ^1^	53	2016	Mature ewes	Singletons and twins	Normal, Group A
16ComB ^1^	59	2016	Mature ewes	Singletons and twins	Normal, Group B
17MatNorm	134	2017	Mature ewes	Twins	Normal
17MatPref	135	2017	Mature ewes	Twins	Supplementary feeding from weaning to breeding
17LambSing	133	2017	Ewe lambs	Singletons	Supplementary feeding from weaning to breeding
17LambTwin	87	2017	Ewe lambs	Twins	Supplementary feeding from weaning to breeding

^1^ The differences between contemporary groups of group A and B were not used in this analysis; ^2^ ewe lambs refer to ewes bred for the first time at eight months of age, and mature ewes refer to three-year-old or older ewes.

**Table 2:** Estimates of variance components and heritabilities with their respective standard errors using the pedigree-based additive genetic relationship matrix and genomic relationship matrix for ewe lamb live weight (LWT) or the occurrence of pregnancy (Preg) when bred for the first time, at eight months of age.

Trait	Pedigree-Based Variance Component Estimates						Genomic Variance Component Estimates			
	$\sigma_{g}^{2}$	$\sigma_{e}^{2}$	$\sigma_{total}^{2}$	$h^{2}$	Correlations		$\sigma_{g}^{2}$	$\sigma_{e}^{2}$	$\sigma_{total}^{2}$	$h^{2}$
LWT	9.73 ± 1.94	7.46 ± 1.55	17.19 ± 0.77	0.57 ± 0.10		$r_{p}$ = 0.19 ± 0.03	9.23 ± 1.53	15.33 ± 1.13	24.56 ± 1.09	0.38 ± 0.05
Preg	0.08 ± 0.03	0.17 ± 0.02	0.40 ± 0.07	0.31 ± 0.11	$r_{g}$ = 0.69 ± 0.14		0.035 ± 0.01	0.22 ± 0.01	0.25 ± 0.01	0.14 ± 0.05

$\sigma_{g}^{2}$ = additive genetic variance; $\sigma_{e}^{2}$ = residual variance; $\sigma_{total}^{2}$ = total phenotypic variance; $h^{2}$ = heritability; $r_{g}$ = genetic correlation between ewe lamb live weight (LWT) and the occurrence of pregnancy (Preg)${;r}_{p}$ = phenotypic correlation between ewe lamb live weight (LWT) and the occurrence of pregnancy (Preg).

**Table 3:** Significant SNPs or nearly significant SNP regions (with the most significant SNP reported) associated with ewe lamb live weight (LWT) or the occurrence of pregnancy (Preg) when bred for the first time at eight months of age, chromosome (Chr), position, effect, standard error of the effect (Effect SE), −log_10_ (*p* value) and gene ^1^.

Trait	Chr	Position	Effect	Effect SE	−log_10_ (*p* Value)	Gene ^1^
LWT	2	9,111,357	−0.722	0.182	4.123808	*TNC*, *TNFSF8*
LWT	4	17,435,731	0.840	0.210	4.183784	*COL28A1*
Preg	1	165,324,517	−0.094	0.025	3.708944	Long non-coding RNA
Preg	2	221,329,561	0.076	0.022	3.366369	*PARD3B*
Preg	3	55,593,107	−0.076	0.021	3.457839	Uncharacterized location ^2^
Preg	4	62,075,731	−0.089	0.024	3.658320	*SMIM30*, *GPR85*
Preg	11	40,677,674	−0.079	0.022	3.460317	*SERPINF1*, *SERPINF2*, *SLC43A2*, *WDR81*, *PRPF8*, *SCARF1*, *INPP5K*, *YWHAE*, *TLCD2*, *RILP*, *MYO1C*, ENSOARG00020010695

^1^ The genes were located within the 150 kb flanking the significant SNP; ^2^ i.e., no annotated gene was associated with the 300 kb area including the nearly significant SNP; *TNC* = tenascin C; *TNFSF8* = TNF superfamily member 8; *COL28A1* = collagen type XXVIII α 1 chain; *PARD3B* = par-3 family cell polarity regulator β; *SMIM30* = small integral membrane protein 30; *GPR85* = G protein-coupled receptor 85; *SERPINF1* = serpin family F member 1; *SERPINF2* = serpin family F member 2; *SLC43A2* = solute carrier family 43 member 2; *WDR81* = WD repeat domain 81; *PRPF8* = pre-mRNA processing factor 8; *SCARF1* = scavenger receptor class F member 1; *INPP5K* = inositol polyphosphate-5-phosphatase K; *YWHAE* = tyrosine 3-monooxygenase/tryptophan-5-monooxygenase activation protein epsilon; *TLCD2* = TLC domain containing 2; *RILP* = Rab interacting lysosomal protein; *MYO1C* = myosin 1C; ENSOARG00020010695 = phosphatidylinositol transfer protein α.

**Table 4:** Trait, gene and gene name, results of the pathway analysis with the Reactome basic function and the targeted pathway analysis, and references from the literature regarding each gene associated with ewe lamb live weight (LWT) at eight months of age.

Trait	Gene	Name	Reactome Basic Function	Targeted Pathway Analysis	Reference
LWT	*TNC*	Tenascin C	Extracellular matrix organization	Implication in adaptative response of musculoskeletal tissues to mechanical stress	[30,31,32]
LWT	*TNFSF8*	TNF superfamily member 8	Immune System	Expressed on activated T cells Regulation of cell survival and apoptosis	[33,34]
LWT	*COL28A1*	Collagen type XXVIII α 1 chain	Extracellular matrix organization	Collagen biosynthesis and modifying enzymes	[35,36]

## Data Availability

The data presented in this experiment are available within the article.
